# Insight of PBZ mediated drought amelioration in crop plants

**DOI:** 10.3389/fpls.2022.1008993

**Published:** 2022-11-29

**Authors:** Chirag Maheshwari, Nitin Kumar Garg, Muzaffar Hasan, Prathap V, Nand Lal Meena, Archana Singh, Aruna Tyagi

**Affiliations:** ^1^ Division of Biochemistry, ICAR-Indian Agricultural Research Institute, New Delhi, India; ^2^ Sri Karan Narendra Agriculture University, Jobner, India; ^3^ Agro Produce Processing Department, Indian Council of Agricultural Research (ICAR)-Central Institute of Agricultural Engineering, Bhopal, India

**Keywords:** PBZ, drought, gibberellic acid, abscisic acid, physiological and biochemical response

## Abstract

Water scarcity is a significant environmental limitation to plant productivity as drought-induced crop output losses are likely to outnumber losses from all other factors. In this context, triazole compounds have recently been discovered to act as plant growth regulators and multi-stress protectants such as heat, chilling, drought, waterlogging, heavy metals, etc. Paclobutrazol (PBZ) [(2RS, 3RS)-1-(4-chlorophenyl)- 4, 4-dimethyl-2-(1H-1, 2, 4-trizol-1-yl)-pentan-3-ol)] disrupts the isoprenoid pathway by blocking ent-kaurene synthesis, affecting gibberellic acid (GA) and abscisic acid (ABA) hormone levels. PBZ affects the level of ethylene and cytokinin by interfering with their biosynthesis pathways. Through a variety of physiological responses, PBZ improves plant survival under drought. Some of the documented responses include a decrease in transpiration rate (due to reduced leaf area), higher diffusive resistance, relieving reduction in water potential, greater relative water content, less water use, and increased antioxidant activity. We examined and discussed current findings as well as the prospective application of PBZ in regulating crop growth and ameliorating abiotic stresses in this review. Furthermore, the influence of PBZ on numerous biochemical, physiological, and molecular processes is thoroughly investigated, resulting in increased crop yield.

## Introduction

Climate change has posed a severe danger to crop productivity and output. Numerous types of abiotic stressors, such as heat, drought, and salt, cause morphological, physiological, and biochemical alterations that eventually hamper crop growth (Yadav et al., 2020). Drought is a big worry since numerous variables such as high and low temperatures, limited water availability, erratic rain patterns, low rainfall, salt, high light intensity, etc. led to drought (Salehi-Lisar and Bakhshayeshan-Agdam, 2016). Drought in plants is characterized by decreased leaf water potential, turgor pressure, stomatal closure, and impaired cell growth. Drought impacts photosynthesis, chlorophyll production, nutrient metabolism, ion uptake and translocation, respiration, carbohydrate metabolism, etc. in plants (Farooq et al., 2009).

When it comes to drought-induced plant damage, oxidative stress is critical. Drought raises reactive oxygen species (ROS) levels in plant cells (Smirnoff, 1993). Excessive ROS generation and accumulation induce cellular oxidative damage, disrupt cellular membranes, and result in enzyme inactivation, protein breakdown, and ionic imbalance in plants (Hasanuzzaman et al., 2020). ROS disrupts cellular macromolecules, including DNA, and may result in base deletion owing to alkylation and oxidation, both of which have been associated with a variety of physiological and biochemical ailments in plants (Apel and Hirt, 2004). Plants have a sophisticated antioxidative defense system that controls the overproduction of ROS. The ROS-induced damages and disruption of cellular homeostasis are alleviated by the action of different enzymatic (e.g., catalase, CAT; superoxide dismutase, SOD; peroxidase, POD; glutathione reductase, GR; glutathione peroxidase, GPX) and non-enzymatic (e.g., ascorbic acid, carotenoids, tocopherols, and glutathione content) antioxidants (Prochazkova et al., 2001; Rady and Gaballah, 2012). This plant defense system is only active up to a specific threshold of tolerance. Under severe and persistent stress, the natural defense system is hampered, resulting in physiological anomalies (Smirnoff, 1993). The mechanism of ROS generation and scavenging by plants with high antioxidative capacity has been linked to plant tolerance to abiotic stressors (Wahid et al., 2014). As a result, various studies have been conducted to improve plant resilience and drought adaptations, as well as to mitigate the negative effects of drought. These studies mostly involve the use of phytoprotectants (such as growth promoters, antioxidant compounds, and osmoprotectants), which are highly effective measures of promoting drought responses in agricultural plants (Garg et al., 2019; Desta and Amare, 2021).

Plant growth regulators (PGRs) are commonly utilized in agriculture to augment overall plant growth. Plant growth regulators have both beneficial and negative effects on growth, development, and plant metabolism (Ashraf et al., 2011; Desta and Amare, 2021). There are several classes of PGR including auxin, abscisic acid, cytokinins, gibberellin, salicylic, jasmonic acid, and ethylene, as well as more recently investigated brassinosteroids, strigolactones, polyamine, and triazole, etc. Due to their intrinsic abiotic stress tolerance inducement through augmenting plant self-defense systems such as antioxidant enzymes and molecules in stress-affected plants, triazole compounds, a class of systemic fungicides, have been investigated to have plant growth promoting properties and are sometimes used as stress-safeguards (Jaleel et al., 2007). Various triazole compounds are used as PGRs such as PBZ, uniconazole, triapenthenol and BAS 111, etc. Triazoles regulate plant growth by changing the balance of key plant hormones i.e., cytokinins, gibberellic, and abscisic acid (Hajihashemi, 2018). Triazoles induce morphological (root growth stimulated and shoot elongation inhibition) and biochemical (enhanced cytokinin synthesis and temporary increase in ABA) changes (Fletcher et al., 2000; Gopi et al., 2007).

PBZ is a triazole compound that plays an important function in reducing water deficit stress by lowering glutathione levels and reducing the peroxidation of lipids (Aly and Latif, 2011). Many plants, including tomato, sesame seeds, and mango have been shown to use PBZ to reduce the negative effects of drought by enhancing the activity of anti-oxidative enzymes (Somasundaram et al., 2009; Srivastav et al., 2010). PBZ has been known to be used in horticultural crops for a long time to increase yield. (Assuero et al., 2012; Kamran et al., 2020). PBZ prevents the biosynthesis of sterol and gibberellins (Khan et al., 2009). By modifying the photosynthetic rate and phytohormone levels, PBZ can significantly affect plant growth and development (Kim et al., 2012) (**Table 1**). The application of PBZ improved leaf number, stem diameter, modified root architecture, decreased plant height, and contributed to enhanced yield and tolerance to lodging (Syahputra et al., 2016; Pal et al., 2016). An enzyme ent-kaurene oxidase in GA biosynthetic pathway which catalyzes ent-kaurene oxidation into ent-kaurene acid is inhibited by PBZ (Rady and Gaballah, 2012; Kondhare et al., 2014). Sankar et al. (2013), reported that PBZ retains endogenous cytokinin levels, stabilizes leaf water capacity, and induces increased leaf and epidermal thickness. Besides discussing possible theories on the regulation of water deficit stress tolerance, this article aims to investigate the impact of PBZ on morphological, biochemical, and molecular responses to drought.

**Table 1:** Summary of effect of PBZ on different parameters under the drought stress in various crops species.

Effect of PBZ on RWC				
S.No.	Crop	Effective dose	Key findings	References
1.	*Triticum aestivum*	30 mg/l	RWC was increased by 5% in control and 11% in drought plants treated with PBZ	Dwivedi et al., 2017
2.	*Oryza sativa*	90 mg/l	15% increase in RWC under drought compared to control	Garg et al., 2019
3.	*Curcuma alismatifolia*	1500 mg/l	RWC increased by 5% under drought	Jungklang et al., 2017
4.	*Abelmoschus esculentus*	80 mg/l	RWC increased by 60.1% under drought	Iqbal et al., 2020
***Effect of PBZ on MSI***				
	*Oryza sativa*	90 mg/l	15% increase in mean MSI under drought	Garg et al., 2019
	*Triticum aestivum*	30 mg/l	4-5% increase in mean MSI under drought	Dwivedi et al., 2017
***Effect of PBZ on plant growth***				
	*Curcuma alismatifolia*	1500 mg/l	The plant height was 1.2 times lower under drought	Jungklang and Saengnil, 2012
	*Curcuma alismatifolia*	3.75 g/l	Shoot height was reduced by 48.93% under drought	Jungklang and Saengnil, 2012
	*Helianthus annuus* and *zinnia*	2.0 mg/pot	Shoot height was reduced by 26.3 and 42.1%, respectively	Ahmad et al., 2014
	*Syzygium myrtifolium*	3.75 g/l	Plant height was reduced by 19.93%	Roseli et al., 2012
	*Curcuma alismatifolia*	1500 mg/l	Plant height was reduced by 50% under drought	Jungklang et al., 2017
	*Odontonema strictum*	0.24 mg/pot	Plants were 11 cm taller under drought	Rezazadeh et al., 2016
	*Amorpha fruticosa*	150 mg/l	61% increase in the plant height	Fan et al., 2020
	*Zea mays* L	300 mg/l	Increased root dry weight by 102.1% at the seventh leaf stage, 65.1% at the ninth leaf stage, 47.9% at the twelfth leaf stage	Kamran et al., 2018
	*Arachis hypogaea*	10 mg/l	Increased root length from 18.17 to 28.15 cm/plant, total leaf area from 96.38 to 117.31 cm^2^/plant, whole plant fresh weight from 33.72 to 39.16 gm/plant, whole plant dry weight from 3.49 to 4.12 g/plant	Sankar et al., 2014
	*Sesamum indicum*	5 mg/l		Abraham et al., 2008
	*Ipomoea batatas*	34 µm	Increased vine fresh weight, root fresh weight, vine dry weight, and root dry weight by 40.10, 65.47, 66.91, and 67.86% respectively	Yooyongwech et al., 2017
***Effect of PBZ on photosynthetic pigments***				
	*Anacardium occidentale*	3 g a.i./tree	Increased Chlorophyll a (27.35%), Chlorophyll b (54.54%), total chlorophyll (30.98%) and Carotenoids (13.55%) under control conditions	Mog et al., 2019
	*Triticum aestivum*	30 mg/l	25.7% increase in chlorophyll content under drought	Dwivedi et al., 2018
	*Zea mays* L	300 mg/l	Increased the chlorophyll content by 48.2%, 54.3%, 51.2% and 79.0%, at 0, 15, 30 and 45 DAS respectively. Carotenoid contents increased by 15.7%, 17.3%, 27.9% and 36.7% at 0, 15, 30 and 45 DAS respectively	Kamran et al., 2020
	*Arachis hypogaea*	10 mg/l	Increased total chlorophyll, carotenoid, xanthophyll and anthocyanin content by 120.22%, 112.66%, 116.48%, 111.26%, 114.44% and 112.24% respectively	Sankar et al., 2013
	*Zea mays* L	2 mg/l	Increased chlorophyll content by 62%	
	*Oryza sativa L. indica*	25 or 50 mg/l	Plants had greener leaves and delayed late senescence	Dewi, 2018
	*Odontonema strictum*	0.24 mg/pot	Net photosynthesis was 51% higher under drought	Rezazadeh et al., 2016
	*Zoysia japonica*	50 mg/l	Increased leaf chlorophyll content by 0.6 mg g^-1^ FW	Cohen et al., 2019
	*Festuca arundinacea* and *Lolium perenne*		Increased the photosynthetic pigment content	Shahrokhi et al., 2011
	*Vigna radiata*	150 mg/l	Increased SPAD value from 34 to 37.7	Babarashi et al., 2021
***Effect of PBZ on grain yield and dry matter partitioning***				
	*Zea mays* L	50 mg/l	Increased the average weight of 1,000 seeds and yield	Bayat and Sepehri, 2012
	*Zea mays* L	300 mg/l	Kamran et al. (2018), average maize grain yields increased by 61.3% after seed soaking with 300 mg/l PBZ, while seed dressing with PBZ at 2.5 g kg^-1^ increased yield by 33.3%	Kamran et al., 2018
	*Triticum aestivum*		Increased grain yield per plant by 6-7%, grain numbers per panicle by 24-33%, 1,000-grain mass by 3-6%, and harvest index by 2-4%	Dwivedi et al., 2017
	*Vigna radiata*	150 mg/l	Increased seed yield from 622 to 1921 kg/ha	Babarashi et al., 2021
	*Odontonema strictum*	0.24 mg/plant	Promoted flowering and maintained the same numbers of flower (6 flowers/plant)	Rezazadeh et al., 2016
	*Solanum lycopersicum*	50 mg/l	Yield increased by 1.37 times more	Rezazadeh et al., 2016
	*Solanum lycopersicum*	30 mg/l	Pretreated tomato plants retained their fruit yield (3.89 kg/plant) and number of fruits (31 fruits/plant) when exposed to drought	Latimer, 1992

## Chemical structure and modes of application

### Chemical structure and translocation of PBZ in plant

**Figure 1:**
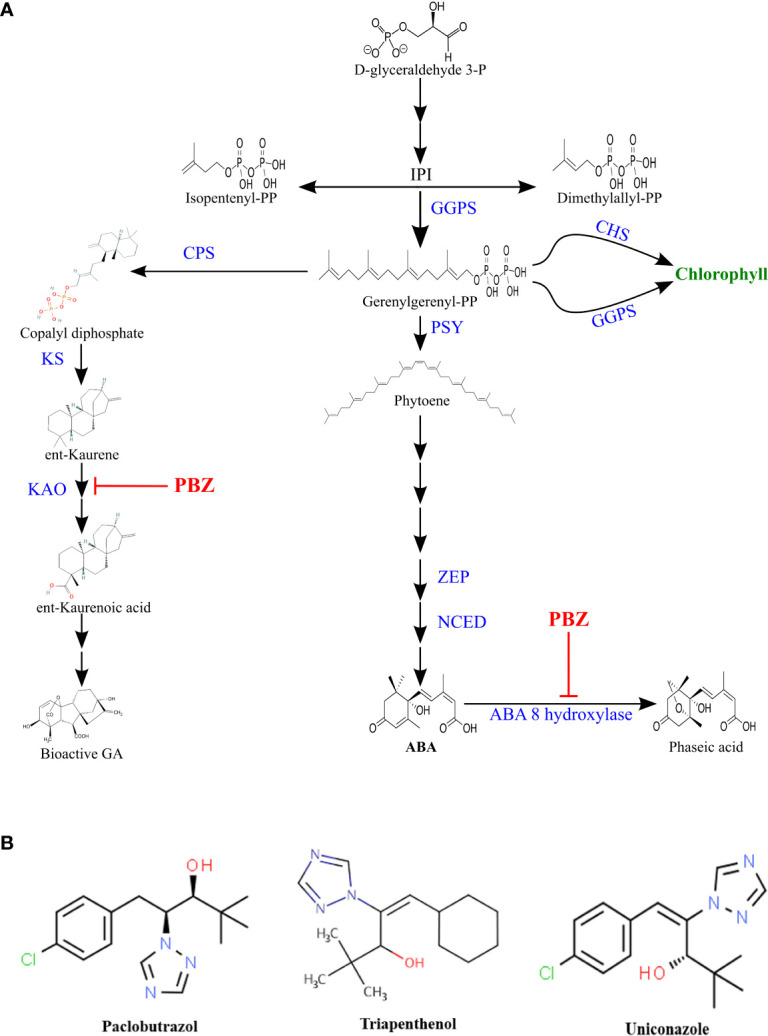
**(A)**Terpenoid pathway. Paclobutrazol inhibition is indicated by 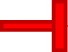. (*CPS*), Copalyl diphosphate synthase; (*KAO*), ent-kaurenoic acid oxidase; (*KS*), ent-kaurene synthase; (*GGPPS*), geranylgeranyl pyrophosphate synthase; (*PSY*), phytoene synthase; (*ZEP)*, zeaxanthin epoxidase; (*NCED*), 9-*cis*epoxycarotenoid dioxygenase; (*CHS*), chlorophyll Synthase; (*GGRS*), geranylgeranyl reductase. **(B)** Chemical structure of some triazoles.

PBZ is a synthetic compound having the empirical formula (1-(4-chloro-phenyl) 4,4-dimethyl-2-(1,2,4-triazol-1-yl)-pentan-3-ol) with two asymmetric carbon (**Figure 1B**). Therefore, two pairs of enantiomers may exist, (2R, 3R) ‐ and (2S, 3S) ‐PBZ, and (2S, 3R) ‐ and (2R, 3S) ‐PBZ. However, due to steric hindrance production of only the first pair of enantiomers is possible (Wu et al., 2013). While in the case of wheat (2S, 3S) ‐PBZ was a more effective regulator of plant growth inhibition than (2R, 3R) ‐PBZ (Lenton et al., 1994).

Triazoles were previously thought to be transported mainly acropetally in the xylem, (Davis et al., 1988). Later research on castor bean (Witchard, 1997) and pear (Browning et al., 1992) found their presence in both xylem and phloem sap, suggesting that these can be transported both acropetally and basipetally. PBZ was also held by roots, translocated through the xylem mainly in the stems, and collected in leaves (Wang et al., 1985). Early and Martin (1988) found that PBZ was metabolized more quickly in apple leaves than in other plant sections.

### Mode of action and methods of application

PBZ is a growth retardant and stress protectant that works by inhibiting GA biosynthesis (Gopi et al., 2009). PBZ suppressed the GA biosynthesis by inactivating ent-kaurene oxidase or cytochrome P450-dependent oxygenase, preventing ent-kaurene to ent-kauronoic acid oxidation (Zhu et al., 2004; Rady and Gaballah, 2012). Since both abscisic acid and chlorophyll are synthesized through the terpenoid pathway, PBZ has been shown to influence their synthesis too (**Figure 1A**). As PBZ inhibits GA synthesis, common terpenoid pathway precursors accumulate and are redirected to promote ABA biosynthesis (Rademacher, 2018). Kitahata et al. (2005) found that PBZ inhibited natural ABA catabolism by inhibiting the ABA 8’ hydroxylase enzyme (Fig 1a).PBZ is more effective even at lower dose of application compared to other PGRs (Rady and Gaballah, 2012).

Foliar sprays, drenching, and seed priming are the most popular methods of PBZ application. All methods yield good results (Rademacher, 2015) but drenching works for a longer duration and provide uniform regulation at lower doses (10 µM) in *Capsicum chinense* (Franca et al., 2017). As PBZ is poorly soluble (0.12 mM) in water when applied as a foliar spray, it is only partially translocated in the phloem (Ribeiro et al., 2011). In contrast to foliar spray, PBZ application by drenching is more uniform as PBZ is transported *via* xylem vessels. Further, PBZ application by drenching inhibits GA more effectively as roots synthesize a significant amount of GA (Sopher et al., 1999). Ruter (1996), demonstrated that drench application was more effective than foliar spray at the lower dose of PBZ (0.5 mg a.i./pot) in shrub lantana. Seed priming treatment using PBZ (100µM) under drought in rice genotypes leads to better growth of the plants compared to unprimed seed plants (Samota et al., 2017a).

## Morphological and physio-biochemical responses of plants to PBZ

### Effect of PBZ on relative water content

Relative water content **(**RWC), directly related to the content of soil water (Sarker et al., 1999) is a significant indicator of water stress in leaves (Merah, 2001). Plant exposure to water stress results in an immediate reduction of RWC (Mawlong et al., 2014; Kumar et al., 2015). PBZ accelerated the stomatal closure, improved water retention, and increased drought tolerance in jack pine and oak ( (Marshall et al., 2000; Percival and Salim AlBalushi., 2007). PBZ-treated plants maintained higher RWC than the non-treated ones’ (Jungklang and Saengnil, 2012; Dwivedi et al., 2017; Jungklang et al., 2017). Dwivedi et al. (2017), stated that the application of PBZ (30 mg/l) in wheat under control and water-stressed plants resulted in an increase of 5% and 11% respectively in the mean RWC. The reduced rate of evapotranspiration helps plants maintain a higher RWC, and overcome stress, and developed tolerance to various environmental stresses (Yadav et al., 2005). RWC increased in PBZ-treated triticale (*Triticale hexaploide*) plants during water stress (Berova and Zlatev, 2003)., Under water stress, PBZ treatment assists plants in retaining water for 30-40 days (Jungklang and Saengnil, 2012). Garg et al. (2019), observed that application of PBZ (90 mg/l) under drought in rice genotypes was responsible for about a 15% increase in RWC as compared to drought without PBZ treatment. Jungklang et al. (2017), found that in *Curcuma alismatifolia* leaves, PBZ (1500 mg/l) increased RWC by 5% under drought. Iqbal et al. (2020), reported that in okra (*Abelmoschus esculentu*) cultivar Nutec, application of PBZ (80 mg/l) along with drought increased RWC (60.1%) compared to drought without PBZ treatment (57.2%) although the result was not statistically significant. Similarly, in Safflower (*Carthamus tinctorius L.*) application of PBZ under drought enhances the RWC (Davari et al., 2022). Overall PBZ enhances the RWC of plants under drought conditions by a reduction in evapotranspiration.

### Effect of PBZ on membrane stability index

Membrane stability is a common criterion for determining drought tolerance because water deficit induces water loss from plant tissues, which severely impairs membrane structure and function. The stability of the cell membrane was used as a drought tolerance indicator and leakage of electrolytes showed an increase in water deficit (Agarie et al., 1995). Garg et al. (2019), reported that PBZ (90 mg/l) in rice genotypes led to an 11% increase in mean MSI as compared to drought-stressed plants without PBZ treatment. PBZ (20 mg/l) minimized the leakage of electrolytes in carrots (Gopi et al., 2007). Dwivedi et al. (2017) reported that the application of PBZ (30 mg/l) in wheat under control and water-stressed plants resulted in an increase of 1-2% and 4-5% respectively in the mean MSI. Similarly, Jungklang et al. (2017), reported that PBZ (1500 mg/l) decreased electrolyte leakage by 60% under water deficit stress in *Curcuma alismatifolia*. Babarashi et al. (2021), observed that the application of PBZ (150 mg/l) in mungbean under drought decreased electrolyte leakage from 52.6% (drought without PBZ) to 47.1%. Similarly, in Safflower (*Carthamus tinctorius L.*) application of PBZ under drought enhances the cell membrane stability (Davari et al., 2022). Collectively, these findings suggest that PBZ improves MSI by minimizing electrolyte and ion leakage under stress conditions.

### Effect of PBZ on plant growth

The most striking growth response observed in PBZ-treated plants is a reduction in shoot growth (Pinto et al., 2005). This response is mainly attributed to internode length reduction. Hua et al. (2014), reported that canola plant height was reduced by 27% when PBZ was applied at 10 cm stalk height as compared to without PBZ. Rezazadeh et al. (2016), reported that red firespike plants treated with PBZ (.24 mg/pot) under drought were 11 cm taller than untreated plants. Under water deficit stress, Jungklang et al. (2017) found that applying PBZ (1500 mg/l) decreased the plant height of *Curcuma alismatifolia* by 50% relative to non-treated plants. In *Amorpha fruticosa*, Fan et al. (2020) found that PBZ treatment (150 mg/l) under extreme drought (RWC 35-40%) resulted in a 61% increase in height relative growth rate compared to drought without PBZ. Jungklang and Saengnil (2012), observed that in Patumma after 40 days of withholding water, the plant height was 1.2 times lower in PBZ (1500 mg/l) treated plants compared to water-stressed without PBZ. When PBZ (3750 mg/L) was applied to Patumma, shoot height was reduced by 48.93% relative to untreated plants. In comparison to non-treated plants, soil drenching with PBZ (1500 mg/l) under water stress for 20- and 30-days periods-maintained shoot length (Jungklang et al., 2017). However, in sunflower and zinnia shoot height was reduced by 26.3 and 42.1%, respectively, after soil drenching with PBZ (2.0 mg/pot) (Ahmad et al., 2014). According to Roseli et al. (2012), *Syzygium myrtifolium* (Roxb.) Walp. plant height was reduced by 19.93% when treated with PBZ (3750 mg/L). According to Azarcon et al. (2022), PBZ (500ppm) increased panicle number, resulting in higher grain yield while reducing water demand, hence increasing rice water use efficiency under drought conditions.

Berova et al. (2002) reported that PBZ (50 mg/l) increased wheat seedling length, fresh and dry weight of shoots, under low-temperature stress as compared to control (low-temperature stress without PBZ). PBZ has been shown to increase both the fresh and dry weight of shoots and roots in cucumber seedlings that have been exposed to high temperatures (Baninasab and Ghobadi, 2011). Kamran et al. (2018), reported that seed soaking of maize with PBZ (300 mg/l) under a semi-arid region increased root dry weight by 102.1% at the seventh leaf stage, 65.1% at the ninth leaf stage, 47.9% at the twelfth leaf stage, compared to drought without PBZ treatment. Sankar et al. (2014), reported that in peanut plants at 80 days after sowing (DAS) application of PBZ (10 mg/l) under drought increased root length from 18.17 to 28.15 cm/plant, total leaf area from 96.38 to 117.31 cm^2^/plant, whole plant fresh weight from 33.72 to 39.16 g/plant, whole plant dry weight from 3.49 to 4.12 g/plant as compared to drought-stressed plants without PBZ treatment. A similar pattern of results was also obtained by Abraham et al. (2008) in *Sesamum indicum* by application of PBZ (5 mg/l) during drought. Yooyongwech et al. (2017) observed that in sweet potatoes, PBZ (34 µM) under drought increased vine fresh weight, root fresh weight, vine dry weight, and root dry weight by 40.10, 65.47, 66.91, and 67.86% respectively, compared to water-stressed plants.

After PBZ (500 mg/l) application, the root dry weight of *Aesculus hippocastanum* was improved (18.4% reduction) after water deficit stress (Percival and Noviss, 2008). Under drought conditions, the dry weight of PBZ (60 mg/l) treated tomato shoots (37.17% reduction) and root dry weight (13.04% reduction) were higher (Latimer, 1992) as compared to the control. Similarly, the dry weight of PBZ (50 mg/l) treated plants decreased by 20.45%, compared to 36.77% for non-treated plants (Bayat and Sepehri, 2012). In turf grass, shoot dry weight was extremely responsive to water deficit conditions (25% FC), resulting in 95 to 97% reduction, respectively, while treatment with PBZ (30 mg/l) reduced the shoot dry weight by 3.14% only (Shahrokhi et al., 2011).

The leaf area of *P. angustifolia* plants treated with PBZ (30 mg/l) and grown under well-watered conditions was reduced by 83.25%. However, when exposed to mild water deficit conditions, the growth of PBZ-treated plants improved but declined when exposed to severe water deficit stress (Fernández et al., 2006). When exposed to drought, shoot height, leaf area, and root length of PBZ (10 mg/l) pre-treated peanut plants improved compared to the control (Sankar et al., 2014a). Farooqi et al. (2010), reported that the diameter of *Vetiveria Zizanioides* increased in stressed plants due to 12% PBZ application. According to Pal et al. (2016), PBZ (1.6 mg/l) reduced leaf area (LA) in tomato plants by 24% under water deficit conditions. Overall, PBZ enhanced plant development under stressful circumstances by increasing shoot and root biomass. Although some research implies that PBZ reduces plant height, others report that PBZ increases plant height, hence a greater knowledge of the influence of PBZ application on plant development is required before future application.

### Effect of PBZ on photosynthetic pigments

Water stress alters the total chlorophyll content and stability within thylakoid membrane protein-pigment complexes which are the first structures to be weakened under stress conditions (Pospíšilová et al., 2000). Chlorophyll reduction under water deficit stress is mainly due to chloroplast damage caused by ROS (Smirnoff, 1995). PBZ (3 g a.i./tree) increased Chlorophyll a (27.35%), Chlorophyll b (54.54%), total chlorophyll (30.98%) and carotenoids (13.55%) compared to control without PBZ in cashew (Mog et al., 2019). According to Dwivedi et al. (2018), applying PBZ (30 mg/l) to wheat plants under water deficit stress resulted in a 25.7% increase in chlorophyll content as compared to stressed plants without PBZ. Kamran et al. (2020), reported that in maize PBZ (300 mg/l) increased the chlorophyll content by 48.2%, 54.3%, 51.2%, and 79.0%, at 0, 15, 30, and 45 DAS respectively Similarly carotenoid contents increased by 15.7%, 17.3%, 27.9% and 36.7% at 0, 15, 30 and 45 DAS in water deficit stress as compared to control (drought without PBZ application). Berova et al. (2002) observed that PBZ treatment was 15–18% more effective than the control at preventing chlorophyll loss in wheat during low-temperature stress. PBZ (10 mg/l) increased total chlorophyll, carotenoid, xanthophyll, and anthocyanin content in 80 days old *Arachis hypogaea* by 120.22%, 112.66%, 116.48%, 111.26%, 114.44%, and 112.24% respectively over control under drought (Sankar et al., 2013) reported that PBZ (2 mg/l) increased chlorophyll content by 62% as compared to control in maize. Dewi et al. (2018), observed that treatment with 25 or 50 mg/l PBZ in black rice plants had greener leaves and encountered late senescence than control plants. Similarly, in Safflower (*Carthamus tinctorius L.*) application of PBZ under drought enhances the photosynthetic pigments (Davari et al., 2022).

Rezazadeh et al. (2016), reported that net photosynthesis was 51% higher in red firespike plants treated with PBZ (0.24 mg/pot) under drought than in those without PBZ. In *Zoysia japonica*, PBZ (50 mg/l) during water deficit stress increased leaf chlorophyll content by 0.6 mg/g FW compared to water-stressed without PBZ (Cohen et al., 2019). Similarly, Pal et al. (2016), recorded that PBZ in both irrigated and deficit-irrigated plants increased chlorophyll content as compared to control plants (without PBZ). PBZ increased the photosynthetic pigment content in *Festuca arundinacea* and *Lolium perenne* under water stress (Shahrokhi et al., 2011). Under water deficit stress, PBZ significantly increased chlorophyll a, chlorophyll b, and carotenoids in wheat cultivars (Aly and Latif, 2011). Babarashi et al. (2021), reported that PBZ (150 mg/l) treatment in mungbean under drought increased SPAD value from 34 (drought without PBZ) to 37.7. All prior investigations have concluded that PBZ improves photosynthesis by increasing chlorophyll and other photosynthetic pigments under stressful circumstances.

### Effect of PBZ on grain yield and dry matter partitioning

Drought primarily affects production by reducing the number of seeds by either influencing the quantity of dry matter produced at the time of flowering or by directly affecting pollen or ovules, leading to a decrease in seed collection. PBZ has been shown to modify sink efficiency, prompting assimilates to be redistributed to meristematic regions other than shoot apices and improving assimilate flow to reproductive structures in plants (Setia et al., 1996). Under drought, the use of PBZ (50 mg/l) increased the average weight of 1,000 seeds and yield in maize (*Zea mays* L.) (Bayat and Sepehri, 2012). According to Kamran et al. (2018), average maize grain yields increased by 61.3% after seed soaking with 300 mg/l PBZ, while seed dressing with PBZ at 2.5 g/kg increased yield by 33.3% compared to control without PBZ in semi-arid regions. Under water stress, wheat genotypes treated with PBZ increased grain yield per plant by 6-7%, grain numbers per panicle by 24-33%, 1,000-grain mass by 3-6%, and harvest index by 2-4% (Dwivedi et al., 2017). According to Iqbal et al. (2020), under water stress, yield per plant was reduced. Stress effects, on the other hand, were found to be reduced when PBZ was applied (40 mg/l). Babarashi et al. (2021) reported that the application of PBZ (150 mg/l) in mungbean under drought increased seed yield from 622 (drought without PBZ) to 1921 kg/ha. Drought impaired flowering in red firespike plants, but PBZ treatment (0.24 mg/plant) promoted flowering and maintained the same number of flowers (6 flowers/plant) as the control (Rezazadeh et al., 2016). Tomato plants treated with PBZ (50 mg/l) produced 1.37 times more fruit than non-treated plants. The yield of pre-treated plants was reduced by 4.79% when they were subjected to drought at 60% field capacity (Mohamed et al., 2011). (Latimer, 1992) observed that PBZ (30 mg/l) pre-treated tomato plants retained their fruit yield (3.89 kg/plant) and fruits per plant (31 fruits/plant) when exposed to water deficit stress. Overall, past research indicates that the use of PBZ boosted grain yield/fruit set under drought by improving sink efficiency.

### PBZ hampered the gibberellin biosynthesis

GAs are growth regulators which fall under a large family of tetracyclic diterpenoids. GAs are plant hormones that are required for a variety of developmental activities in plants such as pollen maturation, stem elongation, leaf expansion, trichome creation, seed germination, and flowering induction (Achard et al., 2009). Furthermore, the exogenous application of gibberellins can reverse PBZ-induced growth inhibition (Lever, 1986). These findings support the theory that PBZ-induced growth inhibition is due to a reduction in gibberellin biosynthesis. Wu et al. (2019), studied the effect of PBZ (200 mg/l) in rice varieties under submergence stress and found that gibberellic acid content was decreased by the application of PBZ compared to submergence stress without PBZ. Fan et al. (2020) found that PBZ (150 mg/l) under severe drought (RWC 35-40%) decreased GA content more than drought without PBZ in *Amorpha fruticosa*.

PBZ-induced abscisic acid biosynthesiAbscisic acid (ABA) is classified as a stress phytohormone because it accumulates quickly in response to stress and mediates many stress responses that help plants survive (Zhang et al., 2006). The effect of PBZ on ABA is of significant importance because ABA is synthesized through the isoprenoid pathway. Fan et al. (2020), reported that PBZ (150 mg/l) under severe drought (RWC 35-40%) increased ABA (27.1%) than without PBZ in *Amorpha fruticosa*. Similarly, Dwivedi et al. (2018), recorded that treatment with PBZ in wheat cultivars did not significantly affect ABA content, however, mean ABA content was significantly enhanced by 25% under water deficit stress. Pal et al. (2016), showed that DI (Deficit irrigated) + PBZ treated plants significantly increased ABA accumulation compared to DI control plants. PBZ application increased ABA and decreased gibberellins during the reproductive stage in the shoot of mango plants (Burondkar et al., 2016). Compared to untreated seedlings, PBZ treatment has been shown to minimize endogenous ABA by about one-third caused by water stress in apples and wheat (Buta and Spaulding, 1991; Wang et al., 2016). Mackay et al. (1990), found that PBZ-induced stress tolerance in snap beans was due to increased endogenous ABA content. PBZ substantially enhanced endogenous ABA levels in hydroponically grown seedlings and detached leaves of oilseed rape, according to Häuser et al. (1990). According to Aly and Latif (2011), PBZ enhanced the endogenous level of ABA in wheat under water deficit stress. Wu et al. (2019), observed that PBZ (200 mg/l) increased ABA content in rice varieties under submergence stress compared to submergence stress without PBZ application. The effect of PBZ on ABA may be the source of stress defense (Fletcher and Hofstra, 1988).

### PBZ elevated antioxidant enzymes activity

PBZ enhances the detoxification of ROS, antioxidant, and chlorophyll (Chl) content (Rady and Gaballah, 2012). As photosystem II (PSII) operation is reduced, an imbalance between electron generation and usage occurs, causing quantum yield shifts. These changes in chloroplastic photochemistry cause excess light energy to be dissipated in the PSII core and antenna under drought, resulting in the development of potentially harmful active oxygen species (O_2_^-1^, 1O_2_, H_2_O_2_, OH) (Peltzer et al., 2002). ROS detoxification pathways can be found in all plant species and are classified as enzymatic which include ascorbate peroxidase (APX), superoxide dismutase (SOD), catalase (CAT), peroxidase (POX), and non-enzymatic which include reduced glutathione (GSH), ascorbic acid and tocopherol (Prochazkova et al., 2001).

Somasundaram et al. (2009) showed that PBZ (5 mg/l) application to *Sesamum indicum* resulted in 464.74%, 267.49%, and 359.08% increase in SOD, APX, and POX activity respectively in leaf tissue under drought conditions as compared to without PBZ. Different PBZ treatments increased SOD activity in maize grown in the semi-arid environment to varying degrees. From 0 to 15 days after silking (DAS), SOD activity increased, then decreased until it reached 45 DAS (Kamran et al., 2020). The APX activity of PBZ-treated ryegrasses was found to be 25% higher than that of untreated under drought. No considerable difference in CAT activity was observed in PBZ-treated plants under drought. PBZ increased POX activity considerably under drought (Sheikh Mohammadi et al., 2017). Under salt stress, a higher dose of PBZ (1500 mg/l) increased the activity of antioxidant enzymes in mango leaves. Under salt stress, mango plants treated with PBZ had higher SOD (24%), POX (163%), and CAT (46%) activity than control plants without PBZ treatment (Srivastav et al., 2010). Application of PBZ increased SOD by 19.09% and 33.07% in roots and leaves, respectively, and CAT activity by 33.17% in quinoa leaves under salinity. Similarly, PBZ improved POD activity in quinoa by 78.18% in roots and 55.56% in leaves under salinity stress (Waqas et al., 2017). Kamran et al. (2020), reported that PBZ (300 mg/l) in semi-arid region increased the mean SOD activity by 12.4%, 22.9%, 29.1%, and 38.6%, POD activity by 21.0%, 33.0%, 32.2% and 59.2%, CAT activity by 29.7, 25.6%, 45.0%, and 70.6%, APX activity by 40.9%, 28.7%, 56.2%, and 53.8% at 0, 15, 30, and 45 DAS, respectively in maize compared with the drought-stressed plants without PBZ treatment. PBZ decreased H_2_O_2_ and O_2_^−^ contents by 51.0% and 40.1% at 0 DAS, 45.0% and 42.0% at 15 DAS, 63.4% and 51.8% at 30 DAS, and 58.2% and 50.4% at 45 DAS, respectively, compared with the water-stressed plants without PBZ treatment. Similarly, Forghani et al. (2020), reported an increase in antioxidant enzymes under salt stress with PBZ in sweet sorghum.

Under water deficit stress, PBZ application resulted in a 2-fold increase in GSH/GSSG ratio compared to control allowing for precise regulation of the ascorbate-peroxidase pathway and, as a result, preventing oxidative damage in tomato plants (Pal et al., 2016). Sankar et al. (2007), reported that in *Arachis hypogaea L.* (ICG221) application of PBZ (10mg/l) under water deficit stress increased ascorbic acid content in leaf from 9.08 to 9.52 mg/g dry weight basis, ά tocopherol from 0.52 to 0.70 mg/g fresh weight basis, reduced glutathione from 33.06 to 47.48 µg/g fresh weight basis as compared to drought-stressed plants without PBZ.

### PBZ enhanced proline content

Proline is a key amino acid in protein and membrane structures, as well as a ROS scavenger under drought (Ashraf and Foolad, 2007). PBZ treatment enhanced proline content and improved drought tolerance. However, further research is needed to determine the actual molecular mechanism underlying the effect of PBZ on mobile proline concentration in plants (Chandra and Roychoudhury, 2020). PBZ treatment (75 mg/L) significantly reduced proline content (0.030 μmol/g FW) in pomegranate leaves by 59.22% to control (0.067 μmol/g FW) (Moradi et al., 2017). Mohamed et al. (2011) found that free proline concentration increased by 54.56 mg g^-1^ in PBZ (50 mg/l) treated tomato plants grown at 60% field capacity, which was 1.52-fold greater than the control. However, in water-stressed conditions, the free proline level in PBZ (10 mg/l) in pre-treated peanuts was lower (1.04-fold over control) than in untreated plants (1.49-fold over control) (Sankar et al., 2014). Dwivedi et al. (2017), showed that the wheat plants treated with PBZ under water stress had a 40% decrease in proline content as compared to the stressed plants without PBZ. These findings suggested that the wheat genotypes experienced less stress (as indicated by the proline content) and improved drought tolerance as a result of PBZ application. Another study showed a considerable increase in free proline content after Mannitol+PBZ treatment in wheat cultivar Sakha 8 (3.342 mg g^-1^ f.w) as compared to control (without PBZ+Mannitol) and the same pattern was observed in all the wheat cultivars (Aly and Latif, 2011). Endogenous proline level increased by 17% in mango leaves treated with PBZ (1500 mg/L) under salt stress when compared to salinized plants without PBZ treatment (Srivastav et al., 2010). Samota et al. (2017a), showed a significant increase in proline content in drought-sensitive and drought tolerant rice genotypes after priming with PBZ under drought as compared to their unprimed samples. Babarashi et al. (2021) reported that the application of PBZ (150 mg/l) in mungbean under drought increased proline content from 7.28 (drought without PBZ) to 7.87 μmol/g f.wt. Similarly, in Safflower (*Carthamus tinctorius L.*) application of PBZ under drought enhances the proline content (Davari et al., 2022).

### PBZ reduced malondialdehyde content

Usually, membrane lipid peroxidation in plants is detected by measuring malondialdehyde (MDA). MDA is a widely used marker of oxidative lipid injury caused by environmental stress. Kamran et al. (2020), showed that the MDA content was significantly lower in the PBZ-treated maize plants over the control under drought. PBZ treatment under drought considerably reduced the MDA content in maize leaf by 31.5% at 0 DAS, 31.4% at 15 DAS, 32.2% at 30 DAS, and 20.2% at 45 DAS compared with drought without PBZ. Other studies carried out on PBZ-primed rice samples indicated that PBZ showed insignificant change in MDA content in the sensitive genotype under drought while a 55% decrease in MDA content was found in the tolerant genotype as compared to PBZ treated under control conditions (Sasi et al., 2021). Similar findings were documented by Samota et al. (2017b), who observed that plants raised from PBZ-primed seeds had lower MDA levels under control and drought conditions than plants raised from unprimed seeds. The amount of MDA decreased as the amount of PBZ increased. PBZ (80 mg/l) decreased MDA content (51.15 mol/g f.wt.) under water deficit stress relative to drought alone (61.92 mol/g f.wt.) (Samota et al. (2017b). Kamran et al. (2020), reported that PBZ (300 mg/l) in the semi-arid region reduced MDA content by 44.1%, 50.4%, 66.3%, 40.5%, at 0, 15, 30, and 45 DAS respectively compared with the water-stressed plants without PBZ treatment.

### PBZ influence on protein content

The protein content in plants decreases with the onset of water deficiency. PBZ treatment increased the protein content of the leaves and tubers in carrots (Gopi et al., 2007). From 0 to 15 DAS, the soluble protein content of maize increased slightly, then steadily decreased from 15 to 45 DAS. Plants treated with a high concentration of PBZ under drought retained higher protein content from 0 to 15 DAS, but protein content was significantly inhibited from 30 to 45 DAS (Kamran et al., 2020). Wheat seeds primed with PBZ had increased protein content (Nouriyani et al., 2012). Also, there are other similar reports which showed that PBZ priming increased the protein content under abiotic stress and non-stress conditions (Razavizadeh and Amu, 2013). According to Iqbal et al. (2020) when PBZ was applied under drought to the okra cultivar Nutec, total soluble proteins increased as the amount of PBZ was increased. Total soluble proteins were 11.04, 11.29, 10.75, and 11.76 mg/g f.wt. at four different PBZ treatments of 0, 20, 40, and 80 mg/l, respectively under water stress conditions.

### PBZ influence on sugar content

During drought, the accumulation of compatible solutes such as carbohydrates is claimed to be an effective stress tolerance mechanism (McKersie and Leshem, 1994). Sugar resulting from transitory starch degradation was noticed in PBZ-pretreated plants (Kaur and Gupta, 1991), which retains the leaf water potential under water deficit stress conditions (Zhu et al., 2004). PBZ treatment in mango increased total sugar, sugar: acid ratio, reducing sugar, and titratable acidity reduction (Vijayalakshmi and Srinivasan, 1999; Yeshitela et al., 2004). In drought-stressed ryegrass, PBZ application significantly increased soluble sugar content compared to untreated plants. The impact of PBZ was mainly pronounced on 30 and 45 days of drought treatment in Iranian perennial ryegrass (Sheikh Mohammadi et al., 2017). According to Fan et al. (2020), PBZ (150 mg/l) under extreme drought (RWC 35-40%) had 119% higher soluble sugar content than drought without PBZ in *Amorpha fruticosa*. In untreated and PBZ-treated (50 mg/l) tomato plants total soluble sugars increased by 1.16 and 1.52 times under water deficit (60% FC), respectively (Mohamed et al., 2011). Sugar content increased by 2 mg/l after foliar application of PBZ under 6% PEG-induced water deficit stress in *S. rebaudiana Bertoni* as compared to stressed plants (Hajihashemi and Ehsanpour, 2014). Total soluble sugar enrichment in PBZ-treated sweet potatoes may be required for cellular osmotic adjustment under water deficit stress situations.

## Molecular responses of plants to PBZ

PBZ inhibits GA biosynthesis by inactivating cytochrome P 450-dependent oxygenase, which inhibits the oxidation of ent-kaurene to ent-kauronoic acid (Zhu et al., 2004; Rady and Gaballah, 2012). PBZ inhibits ABA degradation into phaseic acid, resulting in ABA accumulation. In drought-stressed tomato plants, PBZ increased the expression of ABA biosynthesis genes (SlZEP, SlNCED, and SlAAO1) (Pal et al., 2016). To gain a better understanding of the dwarfism mechanism, Zhu et al. (2016), analyzed gene transcripts of Lily leaves after PBZ treatment. 2704 genes were found to be differentially expressed by comparing PBZ-treated samples to untreated samples. PBZ increased the expression of nine genes encoding GA biosynthesis enzymes (one KAO and eight GA20ox genes) while decreasing the expression of a gene involved in GA deactivation (GA2ox gene). Song et al. (2011) reported that the expression of ent-kaurene oxidase (ZmKO1-2), ent-kaurene synthase (ZmKS1,2,4), and ent-copalyl diphosphate synthase (ZmCPS) decreased, whereas the expression of GA 3-oxidase (*ZmGA3ox1*), GA20-oxidase (*ZmGA20ox1,5*) and ent-kaurenoic acid oxidase (ZmKAO) increased in maize seedlings treated with PBZ. PBZ has been shown to increase SLGA20ox-3 and SLGA3ox2 expression in tomato plants through feedback regulation. Upregulation of SLGA20ox-3 and SLGA3ox2 transcript accumulation was observed in response to PBZ-induced ent-kaurene oxidase inhibition, which was thought to be a feedback upregulation of GA biosynthesis in response to lower GA content (Hedden and Thomas, 2012).

Another study examined the expression profiles of GA biosynthesis genes (ent-kaurene oxidase; *KO*, gibberellin 20-oxidase1; *GA20ox1* and *gibberellin 3-oxidase*; *GA3ox*) and floral transcription factor genes (*UFO*, *WUSCHEL*; *WUS*, and *LFY*) in response to 1,250 mg/l of PBZ treatment of *Jatropha* floral buds. Then, samples were selected at the different time points of 14 days (no sex organs observed), and 20 days after treatment (blooming and sex organs observed). The results showed that PBZ significantly reduced the expression level of *GA20ox1*, *GA3ox*, and *LFY* as compared to the control (*P*<0.05) at 14 days. On the other hand, the expression level of *UFO* and *WUS1* were significantly higher than the control. At 20 days, there was no difference in the expression level of GA biosynthesis genes between the control and treatment. At the same time blooming time of PBZ-treated flowers was delayed which might be due to low expression levels of *GA20ox1*, *GA3ox*, and *LFY* in treated floral buds (Seesangboon et al., 2018).

PBZ (200 mg/l) inhibited the GAs content in rice varieties under submergence stress compared to submergence stress without PBZ (Wu et al., 2019). qRT-PCR was used to analyze the expression of GAs biosynthetic genes such as OsCPS1, OsKS1, and OsGA2ox1. *OsCPS1* mRNA was repressed in PBZ treatment, which was consistent with the GA content in leaves. PBZ application increased ABA content regardless of rice genotypes due to the upregulation of 9-cis-epoxycarotenoid dioxygenase (NCED), the main enzyme in ABA biosynthesis, encoded by OsNCED3 (Barrero et al., 2006).

In contrast to plants not treated with PBZ, Rubisco-small subunit expression was higher at the anthesis and post-anthesis stages in all wheat cultivars with PBZ (Dwivedi et al., 2017). At the anthesis and post-anthesis stages of wheat growth, the PBZ-treated water-stressed plants showed downregulation of the stress marker pyrroline-5-carboxylate synthase (P5CS) expression in all genotypes studied (Dwivedi et al., 2017). At various growth stages after the formation of the basal second internode of wheat, the complex changes in the activities of enzymes involved in lignin biosynthesis, such as phenylalanine ammonia-lyase (PAL) and 4-coumarate: CoA ligase (4CL), were assessed in response to PBZ (200 mg/l) application. The activity of PAL and *4CL* were higher by 42% and 35.6% respectively as compared to the control (Peng et al., 2014; Wang et al., 2016; Kamran et al., 2018).

PBZ (PBZ) at 0.8 and 1.6 mg/l significantly increased aquaporin (gene and protein) expression in tomato plants compared to controls, implying a coordinated increase in ABA and aquaporin levels in response to water stress. Treatment with PBZ during deficit irrigation increased SlTIP2 expression by 5.3-fold above the control and resulted in greater PIP2-7 protein levels (compared to PBZ-irrigated). The increased expression of PIP2-7 in response to PBZ treatment during deficit irrigation shows that it enhances water intake and management by encouraging *de novo* synthesis of aquaporin (AP) channels. Under deficit irrigation, PBZ (0.8 and 1.6 mg/l) administration raised citrate content 2.18 and 1.64-fold, respectively, compared to PBZ-treated irrigated plants (control). This was due to the up-regulation of Sl Citrate synthase (SlCS) by 1.28 and 1.73-folds, respectively. Application of PBZ under irrigated conditions and PBZ-treated deficit irrigated plants increased Sl Succinyl-CoA ligase, SlSCoAL1, and SCoAL2 expression by 1.66 and 2.01-fold, 1.21, and 3.66-fold, respectively, resulting in substantially increased succinate abundance (1.63-fold). PBZ-treated irrigated and deficit irrigated plants produced more GABA than control plants. When PBZ-treated irrigated and deficit irrigated plants were compared to their respective control plants, increased expression of glutamate decarboxylase, SlGAD, was connected to better GABA buildup. GABA production was boosted by increasing the expression of SlGAD, an enzyme necessary for glutamate to GABA conversion.

DNA methylation plays an important role in plant growth and development. Recent research findings have shown that the imposition of various biotic and abiotic stresses on the plant contributes to increased methylation of the genome and thus leads to genome activity degeneration. Garg et al. (2019), found that the application of PBZ under water deficit stress leads to hypermethylation which was predominant in the drought susceptible genotype as compared to drought tolerant genotypes.

## Conclusion

**Figure 2:**
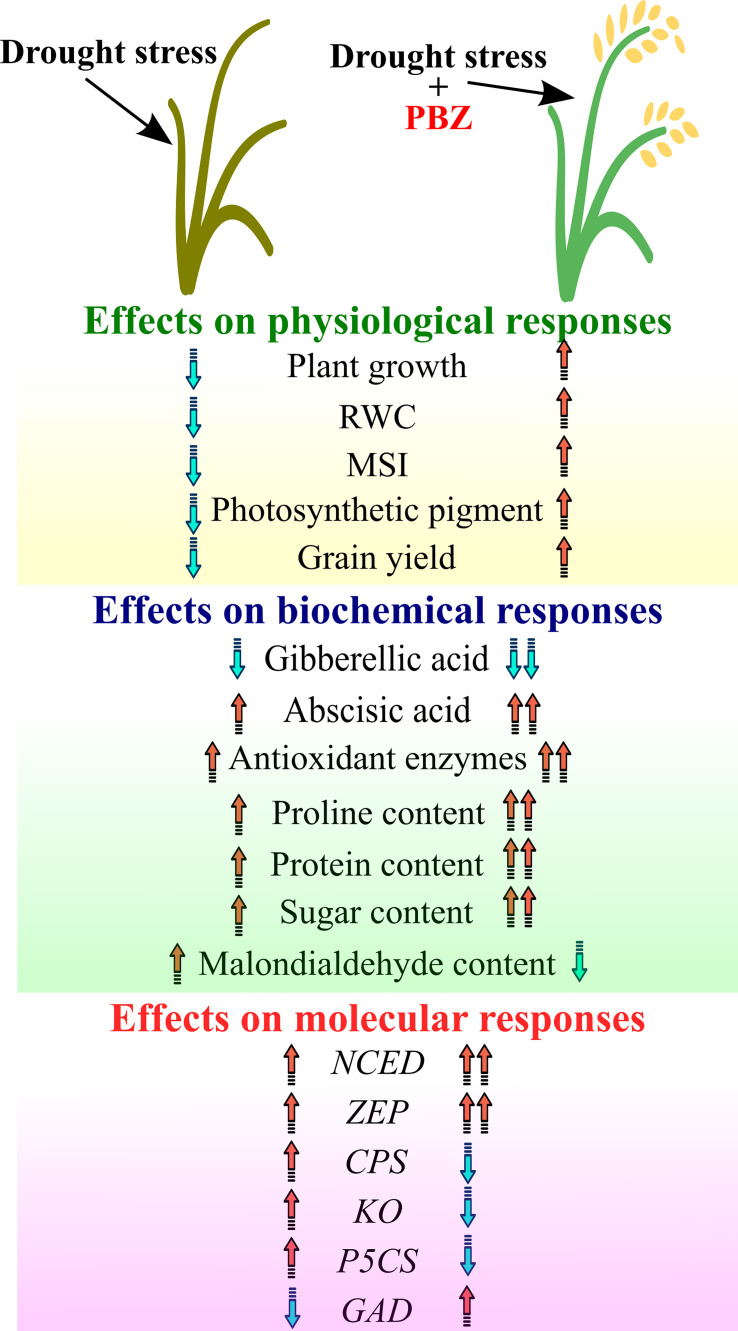
Depiction of overall impact of paclobutrazol under drought on physiological, biochemical, and molecular responses. Arrow showing the trend (Upward-Increase; Downward- Decrease).

By suppressing GA biosynthesis, PBZ increases ABA and chlorophyll. By reducing stomatal conductance and transpiration rates, the increased ABA level increases the RWC and WUE of crop plants. By increasing antioxidant activity and limiting lipid peroxidation, PBZ improved membrane stability and maintained photosynthetic machinery integrity under stress conditions. It also increased the photosynthetic pigment profile, suggesting that the application of PBZ triggers the xanthophyll cycle pigments and thus contributes to the defense of the photosynthetic machinery. As a result, PBZ application increases grain yield by facilitating greater photo assimilation by increasing the exchange of photosynthetic gases, higher chlorophyll content, and photosynthetic activity for longer periods. As a result, PBZ-induced physiological activities boost crop yield under water stress, salinity, temperature stress, and climate change conditions (**Figure 2**), resulting in more sustainable agricultural practices. This, however, is contingent on attracting agricultural scientists’ attention and farmers’ trust in this novel compound in the future. Furthermore, further research is needed to uncover the PBZ-induced multi-stress defense mechanism, especially in terms of its association, interrelation, and crosstalk with other phytohormones and stress-sensitive genes. As PBZ causes many physiological changes as a drought defence mechanism, these changes are not the same in all plant species, so more research is needed to determine the impact of PBZ and its application in crop fields along with its residual impact on soil.

## Author contributions

CM and NKG wrote the manuscript. MH and PV drew figures and tables. CM, NKG, and NLM contributed to collecting the data. CM, NKG, MH, and PV performed editing and helped with tables and figures. AT and AS conceptualized the manuscript. All authors contributed to the article and approved the submitted version.

## Conflict of interest

The authors declare that the research was conducted in the absence of any commercial or financial relationships that could be construed as a potential conflict of interest.

## Publisher’s note

All claims expressed in this article are solely those of the authors and do not necessarily represent those of their affiliated organizations, or those of the publisher, the editors and the reviewers. Any product that may be evaluated in this article, or claim that may be made by its manufacturer, is not guaranteed or endorsed by the publisher.
